# A Prediction Nomogram for Acute Kidney Injury in Very-Low-Birth-Weight Infants: A Retrospective Study

**DOI:** 10.3389/fped.2020.575097

**Published:** 2021-01-15

**Authors:** Qian Hu, Yuan Shi, Zi-Yu Hua, Lei Bao, Fang Li, Hong Wei, Ping Song, He-Jia Ou-Yang, Qiu Li, Mo Wang

**Affiliations:** ^1^Department of Nephrology, Children's Hospital of Chongqing Medical University, Ministry of Education Key Laboratory of Child Development and Disorders, National Clinical Research Center for Child Health and Disorders, China International Science and Technology Cooperation base of Child Development and Critical Disorders, Chongqing Key Laboratory of Child Infection and Immunity, Chongqing, China; ^2^Department of Neonatology, Children's Hospital of Chongqing Medical University, Chongqing, China; ^3^Children's Medical Large Data Intelligent Application as University Engineering Research Center in Chongqing, Chongqing, China; ^4^Information Center, Children's Hospital of Chongqing Medical University, Chongqing, China

**Keywords:** very-low-birth-weight infants, acute kidney injury, predictor, prediction nomogram, prediction model

## Abstract

**Background and objective:** Acute kidney injury (AKI) is recognized as an independent predictor for mortality in very-low-birth-weight (VLBW) infants and is reported to have a high incidence. In this study, we sought to identify the predictors for AKI in VLBW infants and thereby develop a prediction nomogram for the early detection and management of VLBW infants at high risk of developing AKI.

**Methods:** We designed a retrospective study wherein we investigated the baseline hospitalization data of VLBW infants treated at our hospital between January 2012 and October 2018. Independent predictors of AKI in VLBW infants, as identified by multivariate logistic regression, were incorporated into a model. Hosmer–Lemeshow test was used to test the goodness of fit of the model, and a receiver operating characteristic (ROC) curve was plotted to assess the discriminative ability of the model. The model was internally validated using the 10-fold cross-validation method. A nomogram was plotted to predict the risk of AKI in VLBW infants on the basis of the results of multivariate logistic regression analysis.

**Results:** We investigated the data of 604 VLBW infants, of which 144 (23.8%) developed AKI; in 111 (77.1%) of these infants, AKI occurred within 7 days of birth. Multivariate logistic regression analysis identified the following as predictive factors for AKI in VLBW infants: gestational age, red blood cell count within 3 days of birth, serum calcium concentration within 3 days of birth, maternal age of ≥35 years, and pulmonary arterial hypertension or myocardial injury. Furthermore, the nomogram was found to be effective in estimating the risk of AKI in VLBW infants, with an area under the curve (AUC) of 0.794 [95% confidence interval (CI): 0.754–0.834; *P* < 0.001]. Internal validation done by cross-validation showed that the average AUC was 0.788.

**Conclusion:** The nomogram developed in this study was found to be sensitive and specific for the preoperative prediction of AKI in VLBW infants, as per the Kidney Disease: Improving Global Outcomes (KDIGO) criteria modified for neonates.

## Introduction

Acute kidney injury (AKI) is characterized by an abrupt decrease in kidney function (1). AKI is often associated with a poor prognosis in newborns and could result in fatality (2). Very-low-birth-weight (VLBW) infants have underdeveloped kidneys, and they are easily affected by nephrotoxic drugs (3, 4). Therefore, the incidence of AKI has been reported to be high, at 12–40% (5–8), which makes it particularly important to identify the potential predictors for AKI and develop a risk prediction model for the early detection of AKI in VLBW infants (9). Mian et al. retrospectively analyzed the clinical data of a cohort of 266 VLBW infants and found that early gestational age (GA), patent ductus arteriosus (PDA), and prolonged mechanical ventilation were independent predictors for AKI in VLBW infants (10). Al Malla et al. in their retrospective study of 293 VLBW infants, observed that necrotizing enterocolitis (NEC) was an independent predictor for AKI in VLBW infants (5). However, both local and overseas studies have rarely focused on VLBW infants, and no risk prediction model has been established thus far. In recent times, the nomogram is widely used as a simple statistical visual tool for predicting the occurrence, development, and prognosis of various diseases (11–13).

In light of these considerations, we sought to identify the predictors of AKI in VLBW infants in this study and establish a prediction nomogram for the early identification and timely management of AKI in VLBW infants.

## Materials and Methods

### Study Design

This study was planned as a retrospective investigation of the data of all VLBW infants managed at our hospital. The study protocol was approved by the Institutional Review Board of Children's Hospital, Chongqing Medical University, and the approval number is 11/2020.

### Study Population

This study was conducted on the VLBW infants admitted to the Children's Hospital of Chongqing Medical University between January 2012 and October 2018. The criteria for inclusion in this study were age of ≤3 days and birth weight of <1,500 g. Infants were excluded from the study if they met any of the following criteria: (1) severe congenital malformation or inherited metabolic diseases, (2) maternal history of kidney diseases or abnormal renal function, (3) the infant died or was discharged within 3 days of admission, and (4) <2 measurements of serum creatinine levels of the infant during hospitalization. The VLBW infants enrolled in the study were assigned to the case (AKI) group or control (NAKI) group, depending on whether or not they developed AKI.

### Data Collection

Data of the enrolled infants were collected for various parameters: demographic characteristics, such as sex and age; perinatal data, such as 5-min Apgar score; results of laboratory measurements, such as red blood cell (RBC) count and hemoglobin (Hb) concentration, as obtained within 3 days of birth; and accompanying diseases.

### Definitions

The diagnosis of AKI was reconfirmed in each case by applying the Kidney Disease: Improving Global Outcomes (KDIGO) workgroup definition of AKI modified for neonates, which was proposed by Jetton and Askenazi in 2012 (14). The criteria for the diagnosis were as follows: (1) no AKI or stage 0, defined by the absence of change in serum creatinine level or an increase of <0.3 mg/dl; (2) stage 1 AKI, defined by an increase in serum creatinine level by ≥0.3 mg/dl within 48 h or an increase of ≥1.5–1.9 times the reference level within 7 days; (3) stage 2, defined by an increase in serum creatinine level by ≥2–2.9 times the reference level; and (4) stage 3, defined by an increase in serum creatinine level by ≥3 times the reference level or serum creatinine level of ≥2.5 mg/dl or history of dialysis. Serum creatinine concentration was measured with a Cobas 701 automatic biochemical analyzer (Roche, China) using the enzymatic colorimetric method. For each measurement obtained for an infant, the recorded value was compared with the baseline serum creatinine level to determine both the absolute value and the percentage increase from the baseline. The baseline serum creatinine level for each infant was defined as the lowest level recorded previously since the baseline level changes constantly during the first week of birth (15). Infants were diagnosed with pulmonary arterial hypertension (PAH) if any of the following criteria were met (16): (1) estimated systolic pulmonary artery pressure of >35 mmHg, (2) a right-to-left ductus arteriosus shunt, and (3) a right-to-left atrial-level shunt. The diagnosis of myocardial injury was made on the basis of the serum creatine kinase-MB (CK-MB) or serum cardiac troponin I (cTnI) levels, with or without abnormal electrocardiogram evidence (17, 18). Serum CK-MB and cTnI concentrations were measured using an ADVIA Centaur CP automatic chemiluminescence immunoassay analyzer (Siemens Healthineers, China) with a CK-MB reference range of 0.21–5 μg/L, where the CK-MB value of >10 μg/L is consistent with myocardial injury, and a cTnI reference range of 0–0.06 μg/L, where >0.12 μg/L is consistent with myocardial injury.

### Statistical Analysis

All statistical analyses were performed using SAS version 9.4 software. For parameters with continuous data, normal distribution was expressed as mean (standard deviation), and skewed distribution was expressed as median (M) and quartile range (P 25–P 75). Countable data were expressed in rate (%). Data of the various parameters were collected for the AKI and non-AKI (NAKI) groups, and univariate and multivariate logistic regression models were established to explore the predictors related to the occurrence of AKI in VLBW infants. In multivariate logistic regression analysis, we included not only the results of univariate analysis but also the relevant factors identified in clinical experience. Forward stepwise regression analysis was performed to screen for independent variables. The Hosmer–Lemeshow test was used to evaluate the goodness of fit of the model. Based on the predicted scores obtained from the multivariate model, a receiver operating characteristic (ROC) curve was plotted to assess the discriminative ability of the model. Area under the curve (AUC) with 95% confidence interval (CI) and the associated *P*-value were calculated. By maximizing Youden Index (i.e., sensitive + specificity – 1), the sensitivity and specificity of the prediction model were also determined. The model was internally validated using the 10-fold cross-validation method. A nomogram was plotted to predict the risk of AKI in VLBW infants on the basis of the results of multivariate logistic regression model.

## Results

### General Information

**Figure 1:**
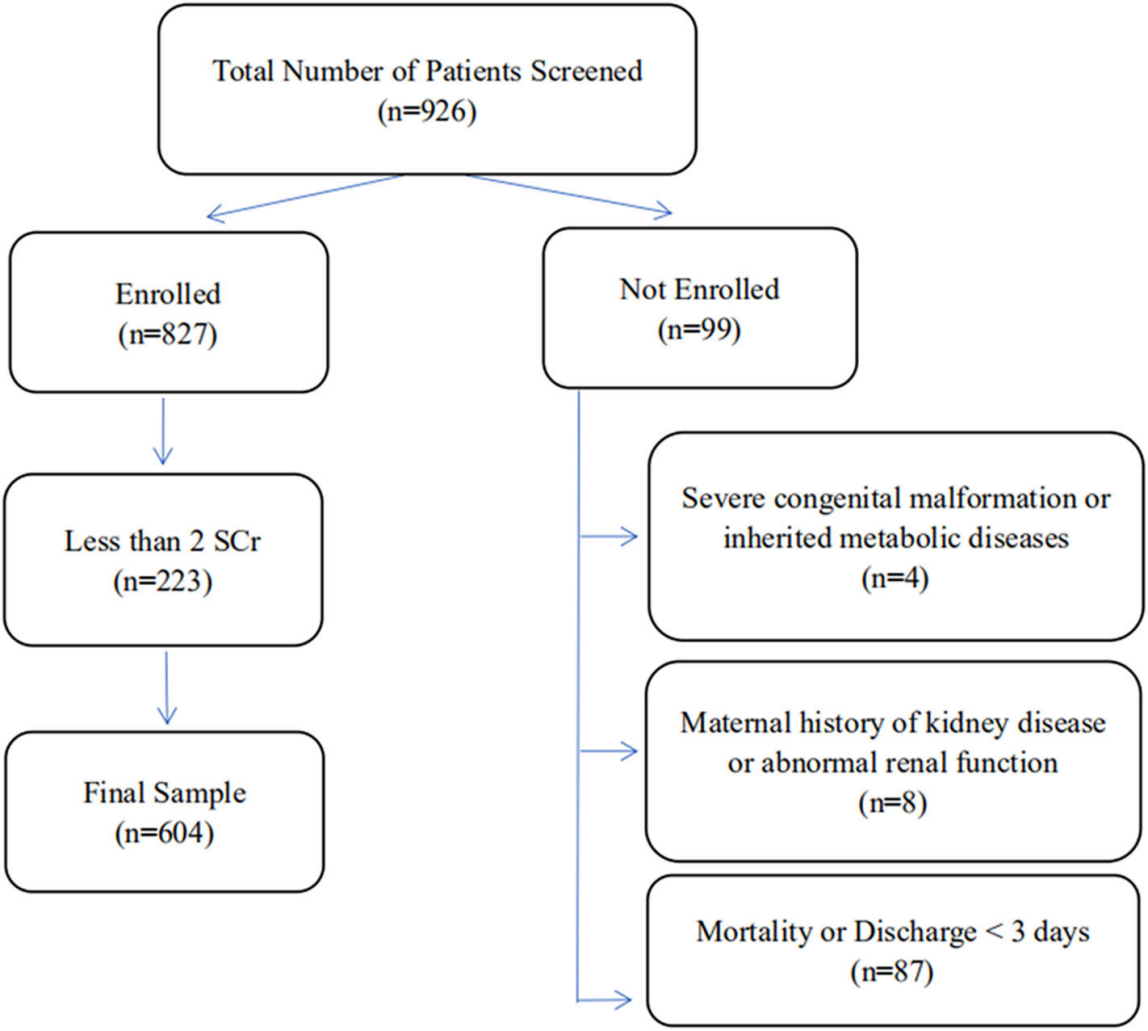
Flowchart of patients enrolled and excluded from the study.

In all, 926 VLBW infants of age ≤3 days were treated at our center during the study period. Of these, 322 were not enrolled since they met one or more of the exclusion criteria, and 604 VLBW infants were ultimately included in this study (Figure 1). According to the modified KDIGO criteria for neonates, 144/604 (23.8%) VLBW infants were included in the AKI group, including 83 males (57.6%) and 61 females (42.4%) (Table 1). In 111/144 (77.1%) cases, signs of AKI were detected within 7 days of birth (Table 2). In all cases of AKI, the condition only occurred once during hospitalization. Among them, 64/144 (44.4%), 51/144 (35.4%), and 29/144 (20.1%) had AKI of stage 1, stage 2, and stage 3, respectively (Table 3).

**Table 1:** Demographic data of the patients.

**Variables**	**(*n* = 144)**	**AKI**	**NAKI**	**Total**
		**(*n* = 460)**	**(*n* = 604)**	
Age (h)	*N*	144	460	604
	Median (range)	2.4 (0.5, 70.3)	2.3 (0.3, 72)	2.3 (0.3, 72)
Gestational age (week)	*N*	144	457	601
	Median (range)	29.1 (25.1, 36.3)	30.9 (24.9, 40.3)	30.3 (24.9, 40.3)
Birth weight (g)	*N*	144	460	604
	Median (range)	1,240 (600, 1,490)	1,320 (550, 1,495)	1,300 (550, 1,495)
Apgar 5	*N*	137	437	574
	Median (range)	8 (2, 10)	9 (1, 10)	9 (1, 10)
Maternal age (year)	<35 years	115 (79.9%)	385 (84.1%)	500 (83.1%)
	≥35 years	29 (20.1%)	73 (15.9%)	102 (16.9%)
Sex of infant	Female	61 (42.4%)	238 (51.7%)	299 (49.5%)
	Male	83 (57.7%)	222 (48.3%)	305 (50.5%)
Type of delivery	Normal delivery	95 (66.0%)	220 (47.9%)	315 (52.2%)
	Cesarean section	49 (34.0%)	239 (52.1%)	288 (47.8%)
RBC (×10^12^/L)	*N*	143	459	602
	Mean (SD)	4.1 (0.7)	4.4 (0.7)	4.4 (0.7)
Hb (g/L)	*N*	143	459	602
	Median (range)	330 (284.5, 368.5)	336 (283, 411)	334.5 (283, 411)
UN (mmol/L)	*N*	144	458	602
	Median (range)	3.9 (1, 12.3)	3.7 (1.1, 19.6)	3.7 (1, 19.6)
CysC (mg/L)	*N*	35	76	111
	Mean (SD)	2 (0.4)	1.9 (0.3)	1.9 (0.3)
P (mmol/L)	*N*	144	459	603
	Median (range)	2.2 (1, 5.4)	2.1 (0.8, 4.3)	2.1 (0.8, 5.4)
Ca (mmol/L)	N	144	459	603
	Median (range)	1.9 (0.9, 2.5)	2.1 (1, 2.8)	2 (0.9, 2.8)
Respiratory failure	–	22 (15.5%)	165 (36.7%)	187 (31.6%)
	+	120 (84.5%)	285 (63.3%)	405 (68.4%)
Sepsis	–	54 (38.0%)	235 (52.2%)	289 (48.8%)
	+−	20 (14.1%)	63 (14%)	83 (14.0%)
	+	68 (47.9%)	152 (33.8%)	220 (37.2%)
Brain injury	–	110 (77.5%)	385 (85.6%)	495 (83.6%)
	+	32 (22.6%)	65 (14.4%)	97 (16.4%)
Pulmonary hypertension	–	67 (47.2%)	336 (74.7%)	403 (68.1%)
	+	75 (52.8%)	114 (25.3%)	189 (31.9%)
PDA	–	33 (23.2%)	213 (47.3%)	246 (41.6%)
	+	109 (76.8%)	237 (52.7%)	346 (58.5%)
Myocardial injury	–	46 (32.4%)	217 (48.2%)	263 (44.4%)
	+	96 (67.6%)	233 (51.8%)	329 (55.6%)
Neonatal hyperbilirubinemia	–	31 (21.8%)	137 (30.4%)	168 (28.4%)
	+	111 (78.2%)	313 (69.6%)	424 (71.6%)
NEC	–	111 (78.2%)	369 (82.0%)	480 (81.1%)
	+−	19 (13.4%)	51 (11.3%)	70 (11.8%)
	+	12 (8.5%)	30 (6.7%)	42 (7.1%)
Neonatal asphyxia	–	77 (53.47%)	282 (61.3%)	359 (59.44%)
	+	65 (45.14%)	168 (36.52%)	233 (38.58%)

*+, yes; –, no; +−, suspected*.

*AKI, acute kidney injury; NAKI, non-acute kidney injury; N, number; Apgar 5, 5-min Apgar score; RBC, red blood cell count within 3 days of birth; Hb, hemoglobin concentration within 3 days of birth; UN, urea nitrogen within 3 days of birth; CysC, serum cystatin C within 3 days of birth; P, phosphorus concentration within 3 days of birth; Ca, calcium concentration within 3 days of birth; PDA, patent ductus arteriosus; NEC, necrotizing enterocolitis*.

**Table 2:** AKI stage.

**Stage**	**Number (%)**
1	64 (44.4)
2	51 (35.4)
3	29 (20.1)
Total	144 (100.0)

*AKI, acute kidney injury*.

**Table 3:** Time of occurrence of AKI.

**Time (day)**	**Number (%)**
<8	111 (77.1)
8–20	30 (20.8)
>20	3 (2.1)
Total	144 (100.0)

*AKI, acute kidney injury*.

### Differences in Clinical Manifestations

Compared with the NAKI group, the AKI group had more male infants and cases of natural delivery and lower values of GA, birth weight, and 5-min Apgar score. Furthermore, when compared with the NAKI group, the AKI had lower levels of RBC count, Hb concentration, and serum calcium (Ca) concentration, but higher level of serum phosphorus (P) concentration, at 3 days of birth. As compared with the NAKI group, the AKI group had higher incidences of comorbidities, such as respiratory failure, sepsis, brain injury, PAH, PDA, myocardial injury, and neonatal hyperbilirubinemia (Table 1).

### Univariate Regression Analysis

Univariate regression analysis of the clinical data of 604 VLBW infants revealed that the following were significantly associated with the risk of AKI occurring in VLBW infants: GA; type of delivery; birth weight; 5-min Apgar score; RBC count, Hb concentration, Ca concentration, and P concentration within 3 days of birth; and history of respiratory failure, sepsis, brain injury, PAH, PDA, myocardial injury, or neonatal hyperbilirubinemia (Table 4).

**Table 4:** Univariate and multivariate logistic regression analyses of predictors for AKI in VLBW infants.

**Variables**		**Univariate**			**Multivariate**		
		**Coefficient**	**OR (95% CI)**	***P***	**Coefficient**	**OR (95% CI)**	***P***
Age (h)		−0.003	0.997 (0.979, 1.015)	0.738			
Gestational age (week)		−0.332	0.717 (0.649, 0.793)	<0.001	−0.289	0.749 (0.67, 0.837)	<0.001
Birth weight (g)		−0.017	0.983 (0.973, 0.993)	0.001			
Apgar 5		−0.155	0.857 (0.777, 0.945)	0.002			
RBC (×10^12^/L)		−0.703	0.495 (0.374, 0.656)	<0.001	−0.395	0.673 (0.485, 0.935)	0.018
Hb (g/L)		−0.274	0.761 (0.669, 0.865)	<0.001			
UN (mmol/L)		0.013	1.014 (0.943, 1.09)	0.715			
CysC (mg/L)		1.153	3.166 (0.966, 10.379)	0.057			
P (mmol/L)		0.782	2.185 (1.501, 3.181)	<0.001			
Ca (mmol/L)		−2.038	0.13 (0.065, 0.263)	<0.001	−1.118	0.327 (0.144, 0.743)	0.008
Maternal age	<35 years		1.000				
	≥35 years	0.285	1.33 (0.825, 2.145)	0.242	0.555	1.741 (1.005, 3.017)	0.048
Sex	Male		1.000				
	Female	−0.378	0.686 (0.47, 1)	0.05			
Type of delivery	Normal delivery		1.000				
	Cesarean delivery	−0.745	0.475 (0.321, 0.701)	<0.001			
Respiratory failure	–		1.000				
	+	1.150	3.158 (1.928, 5.172)	<0.001			
Neonatal septicemia	–		1.000				
	+−	0.323	1.382 (0.771, 2.476)	0.278			
	+	0.666	1.947 (1.29, 2.938)	0.002			
Brain injury	–		1.000				
	+	0.544	1.723 (1.073, 2.766)	0.024			
Pulmonary hypertension	–		1.000				
	+	1.194	3.299 (2.229, 4.883)	<0.001	1.030	2.8 (1.805, 4.343)	<0.001
PDA	–		1.000				
	+	1.088	2.969 (1.929, 4.569)	<0.001			
Myocardial injury	–		1.000				
	+	0.665	1.944 (1.307, 2.891)	0.001	0.596	1.816 (1.156, 2.852)	0.01
Neonatal hyperbilirubinemia	–		1.000				
	+	0.449	1.567 (1.003, 2.447)	0.048			
NEC	–		1.000				
	+−	0.214	1.238 (0.702, 2.185)	0.46			
	+	0.285	1.33 (0.659, 2.684)	0.426			
Neonatal asphyxia	–		1.000				
	+	0.349	1.417 (0.968, 2.075)	0.073			

*+, yes; –, no; +−, suspected*.

*AKI, acute kidney injury; VLBW, very-low-birth-weight; OR, odds ratio; Apgar 5, 5-min Apgar score; RBC, red blood cell count within 3 days of birth; Hb, hemoglobin concentration within 3 days of birth; UN, urea nitrogen within 3 days of birth; CysC, serum cystatin C within 3 days of birth; P, phosphorus concentration within 3 days of birth; Ca, calcium concentration within 3 days of birth; PDA, patent ductus arteriosus; NEC, necrotizing enterocolitis*.

### Multivariate Logistic Regression Analysis and Establishment of Risk Prediction Nomogram

Multivariate logistic regression analysis included not only the results of univariate analysis but also the relevant factors identified in clinical experience, such as sex, birth weight, maternal age, and blood urea nitrogen concentration within 3 days of birth. The following were found to be independent predictors for AKI in VLBW infants: GA, maternal age of ≥35 years, low RBC count within 3 days of birth, low Ca concentration within 3 days of birth, and history of PAH or myocardial injury (Table 4). By setting the occurrence of AKI in VLBW infants as 1 and no occurrence of AKI as 0, we were able to establish a multivariate logistic regression model as follows:

$$\begin{array}{l} \text{Log }\left(\text{odds of AKI}\right)=10.616-0.289\;\times \text{ GA }-\;0.395\;\times \text{ RBC}\\ -1.118\;\times \text{ Ca }+\;0.555\times \text{ MA }\geq 35+1.030\;\times \text{ PAH }+\;0.596\;\times \text{ MI} \end{array}$$

where: AKI = acute kidney injury, GA = gestational age, RBC = red blood cell count within 3 days of birth, Ca = serum calcium concentration within 3 days of birth, MA = maternal age, PAH = history of pulmonary arterial hypertension, MI = history of myocardial injury.

**Figure 2:**
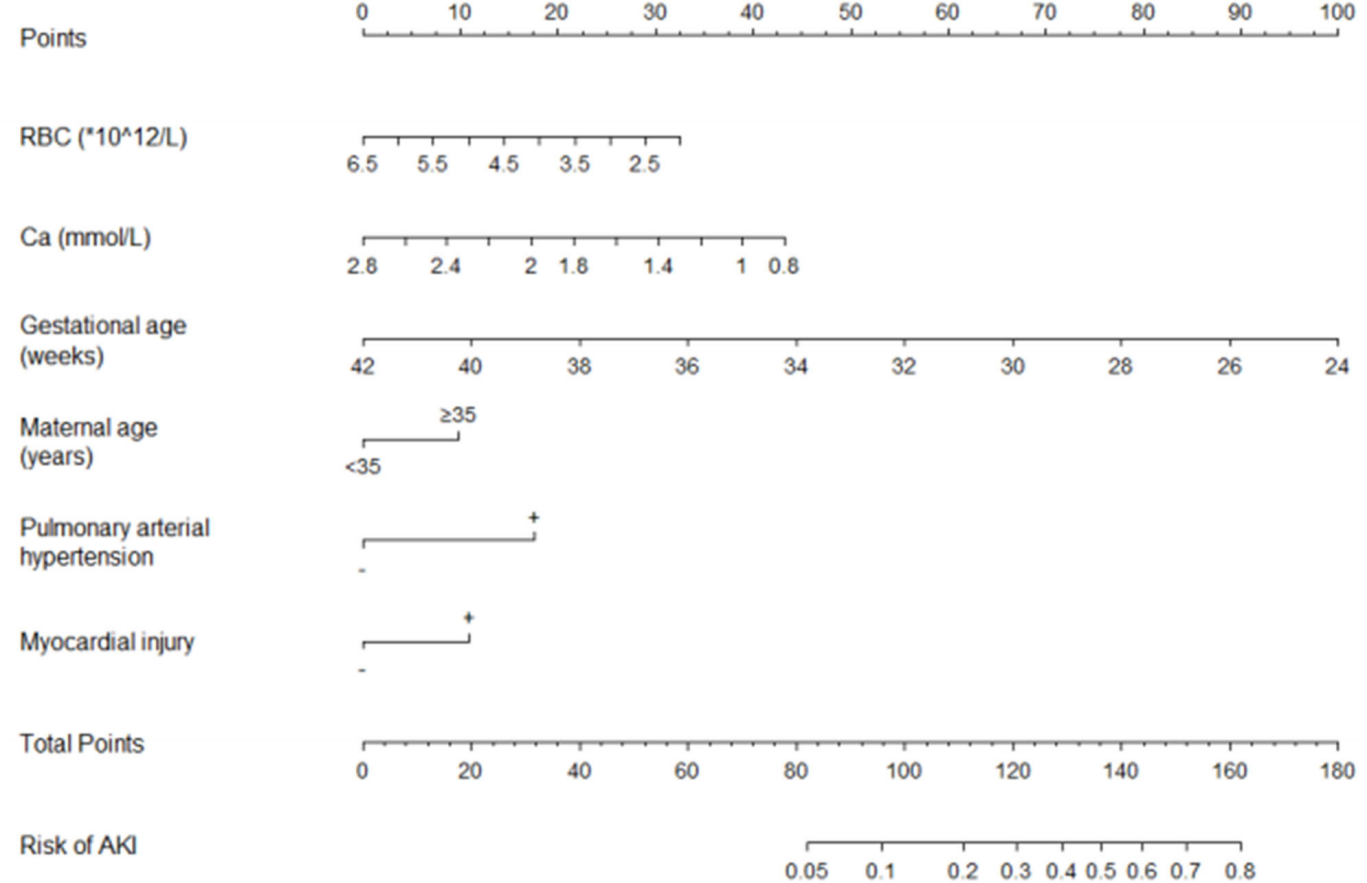
The nomogram for the prediction of AKI in VLBW infants. AKI, acute kidney injury; VLBW, very-low-birth-weight; RBC, red blood cell count within 3 days of birth; Ca, calcium concentration within 3 days of birth.

We performed multivariate logistic regression analysis to establish a nomogram for the prediction of AKI in VLBW infants and scored the identified factors according to their regression coefficient (Figure 2). If an infant has a RBC count of 2 × 10^12^/L within 3 days of birth, the corresponding score is approximately 32.5. If the infant's serum Ca concentration within 3 days of birth is 1 mmol/L, the corresponding score is about 38.8. If the infant's GA is 30 weeks, the corresponding score is about 66.9. If the maternal age is ≥35 years, the corresponding score is about 10. If the infant is diagnosed with PAH, the score is about 17.6. If the infant has no myocardial injury, the corresponding score is 0. In this case, the total score would be 165.8, which indicates a high risk of AKI (>0.8). Therefore, the risk of AKI in this infant is high. Accordingly, the parents of the infant must be informed in time, and early intervention is warranted.

**Figure 3:**
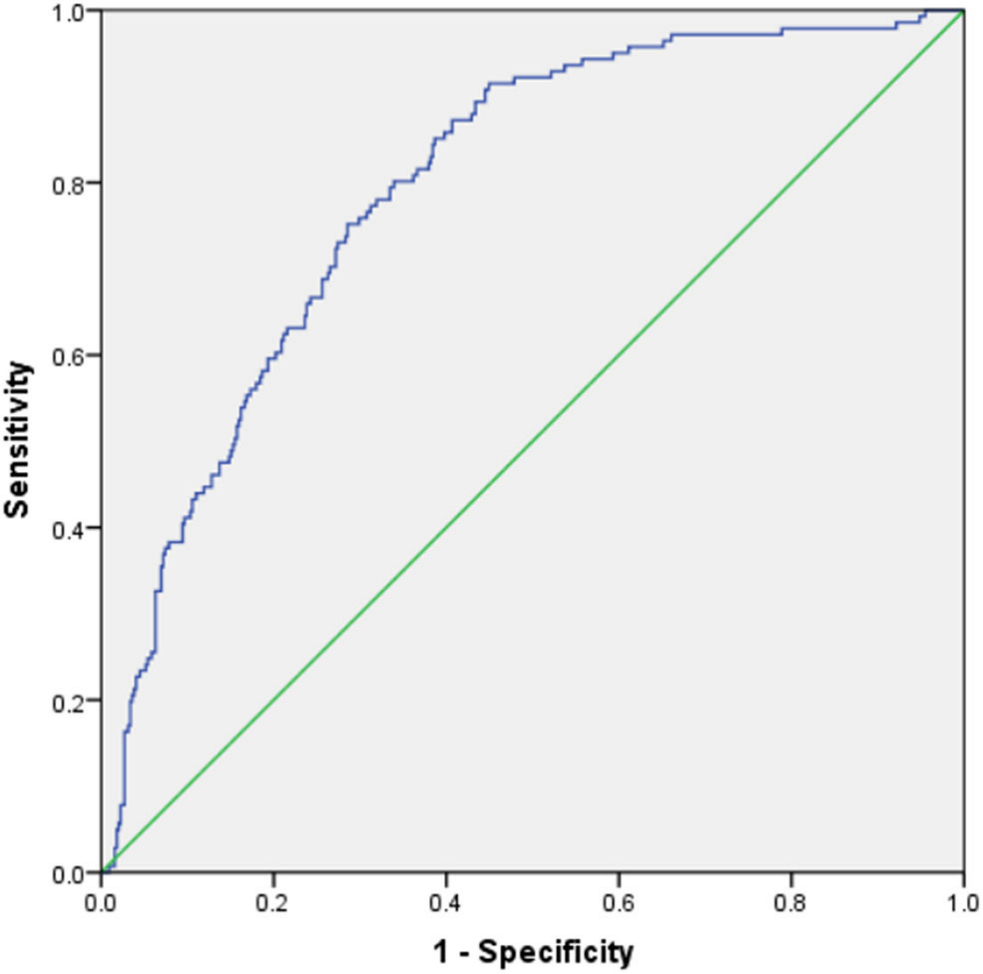
The ROC curve of the model forecasting the occurrence of AKI in VLBW infants.

The Hosmer–Lemeshow test showed that the model was a good fit (P = 0.245). ROC curve analysis of the risk prediction model for AKI in VLBW infants is shown in Figure 3. Sensitivity of the model was 0.752, whereas specificity was 0.715 (AUC = 0.794; 95% CI = 0.754, 0.834; *P* < 0.001). Internal validation showed that the average AUC was 0.788 (Table 5).

**Table 5:** Cross-validation for internal validation.

**Fold**	**1**	**2**	**3**	**4**	**5**	**6**	**7**	**8**	**9**	**10**	**Average**
AUC	0.771	0.883	0.772	0.832	0.794	0.764	0.791	0.809	0.706	0.754	0.788

*AUC, area under the curve*.

## Discussion

AKI is recognized as an independent predictor for mortality in neonates, especially in VLBW infants. Even a brief or mild episode of AKI in infancy puts the surviving infants at great risk of subsequently developing chronic kidney disease. Therefore, the early detection of AKI and timely intervention are of paramount importance (4, 19–21).

In this study, we employed the modified KDIGO definition for the diagnosis of AKI (14). Due to the burden of data collection, we did not use urine output as a diagnostic criterion for AKI in this study.

In this study, we retrospectively investigated 604 VLBW infants and found that most VLBW infants developed AKI within 7 days of birth, and that the occurrence of AKI was related to multiple factors. These findings are consistent with those reported by Carmody et al. (8) and Jetton et al. (22).

This study revealed that early GA was significantly associated with an increased risk of AKI in VLBW infants. This finding is consistent with the observations by Carmody et al. (8) and Mian et al. (10). This may be attributed to the fact that the earlier the GA, the lesser is the number of nephrons and their maturity (23), which leads to an increased susceptibility toward kidney injury (24).

In our study, we observed that maternal age of ≥35 years was an independent predictor for AKI in VLBW infants; this finding is consistent with that by Matyanga et al. (25). However, so far, there is no clear evidence to explain this phenomenon. In agreement with the findings by Ghobrial et al. regarding the predictors of neonatal AKI (26), our findings indicated that PAH was an independent predictor for AKI in VLBW infants. This may be attributed to the fact that PAH leads to circulatory disturbance, which may, in turn, lead to decreased renal perfusion and, eventually, prerenal AKI (27). Chen et al. have shown that myocardial injury is associated with the risk of AKI in patients attempting suicide by charcoal burning (28); similarly, we found that myocardial injury is an independent predictor for AKI in VLBW infants. A possible reason for this is that myocardial injury leads to the instability of hemodynamics, which is associated with prerenal AKI (27). For the first time, we were able to show that a low RBC count within 3 days of birth was associated with a significantly high risk of AKI in VLBW infants. The possible explanation for this increase is that a low RBC count in VLBW infants may increase their susceptibility to hypo-oxygenation of kidney tissue on reduced renal perfusion, which in turn could lead to decreased function. In this study, we observed, for the first time, that a low serum Ca concentration within 3 days of birth is an independent predictor for AKI in VLBW infants. This may be explained by the fact that a decrease in serum Ca concentration could decrease the cardiac output (29), which may be associated with decreased renal perfusion and prerenal kidney injury (27).

In this study, we explored the potential predictors for AKI in VLBW infants and developed, for the first time, a risk prediction model for the early identification and management of AKI in VLBW infants. The Hosmer–Lemeshow test showed that the model was a good fit. A discriminative ability test performed by plotting the ROC curve showed that the sensitivity and specificity of the model were 0.752 and 0.715, respectively (AUC = 0.794; 95% CI = 0.754, 0.834; *P* < 0.001). Internal validation by the cross-validation method showed that the model performed well in our cases. These findings together indicate that this model is highly accurate in predicting AKI in VLBW infants. We then used a nomogram to visualize the model, thereby making it simple and intuitive for practical application.

## Limitations

Studies by Ghobrial et al. (26) and Zhang et al. (30) have shown that there is a close relationship between the occurrence of AKI in neonates and the levels of blood urea nitrogen and serum cystatin C. However, in our study, we did not find any significant difference between the levels of blood urea nitrogen and serum cystatin C in infants with or without kidney injury. With regard to serum cystatin C level, this may be due to the unavailability of sufficient data on serum cystatin C levels at our center. On the other hand, with respect to blood urea nitrogen, the level was higher in the case group than in the control group, but the difference was not statistically significant. This lack of significant difference may be explained as follows: blood urea nitrogen is less sensitive than serum creatinine (31), and about 80% of cases with AKI in this study were in AKI stage 1 or stage 2 (Table 2). Since the nomogram established in this study is based on a retrospective study, further investigations based on data from multiple centers are still necessary to verify our findings and improve the model. Furthermore, our model was evaluated only for infants whose laboratory test data within 3 days of birth were available; further investigations are warranted to confirm the widespread applicability of our findings.

## Conclusion

In this study, we found that GA, RBC count within 3 days of birth, serum calcium concentration within 3 days of birth, maternal age of ≥35 years, and history of PAH or myocardial injury were predictors for the occurrence of AKI in VLBW infants. We developed a nomogram based on these findings and found that it performed well. We intend to undertake external validation studies to verify the predictive accuracy of this model in diverse patient populations. Further refinement of this model would be possible by including analysis for factors, such as serum cystatin C and neutrophil gelatinase-associated lipocalin.

## Data Availability Statement

The raw data supporting the conclusions of this article will be made available by the authors, without undue reservation.

## Ethics Statement

The studies involving human participants were reviewed and approved by the Institutional Review Board of Children's Hospital, Chongqing Medical University. Written informed consent to participate in this study was provided by the participants' legal guardian/next of kin.

## Author Contributions

QH contributed to the study concept and design, data interpretation, literature review, and manuscript writing. YS contributed to the study concept and design, data interpretation, and literature review. Z-YH, LB, FL, HW, and QL contributed to the data interpretation and literature review. PS and H-JO-Y contributed to the data collection and statistical analysis. MW implemented and led the study. All authors contributed to the manuscript revision and have read and approved the submitted version.

## Conflict of Interest

The authors declare that the research was conducted in the absence of any commercial or financial relationships that could be construed as a potential conflict of interest.
